# Why is repositioning public health innovation towards a social paradigm necessary? A reflection on the field of public health through the examples of Ebola and Covid-19

**DOI:** 10.1186/s12992-021-00695-3

**Published:** 2021-04-14

**Authors:** Marietou Niang, Sophie Dupéré, Hassane Alami, Marie-Pierre Gagnon

**Affiliations:** 1 Faculty of Nursing Science, Université Laval, 1050, Avenue de la Médecine, Pavillon Ferdinand-Vandry, Québec, QC G1V 0A6 Canada; 2 Center for Public Health Research, Université de Montréal, Montreal, Québec Canada

**Keywords:** Health innovation, Technological innovation, Social innovation, Public health, Global health, Ebola, Covid-19

## Abstract

Health innovations are generally oriented on a techno-economic vision. In this perspective, technologies are seen as an end in themselves, and there is no arrangement between the technical and the social values of innovation. This vision prevails in sanitary crises, in which management is carried out based on the search for punctual, reactive, and technical solutions to remedy a specific problem without a systemic/holistic, sustainable, or proactive approach. This paper attempts to contribute to the literature on the epistemological orientation of innovations in the field of public health. Taking the Covid-19 and Ebola crises as examples, the primary objective is to show how innovation in health is oriented towards a techno-economic paradigm. Second, we propose a repositioning of public health innovation towards a social paradigm that will put more emphasis on the interaction between social and health dimensions in the perspective of social change. We will conclude by highlighting the roles that public health could play in allowing innovations to have more social value, especially during sanitary crises.

## Background

An innovation is often defined as any “idea, knowledge, technology, product, policy, process or practice perceived as new by the individual or the adoption unit” [1]. Innovation is imperative to stimulating growth [2] or creating positive change, especially during crises, which are favorable and opportune moments to innovate [3]. A close link exists between innovation, health, productivity and wealth, but understanding of this relationship is limited. In fact, there is a belief that the most innovative countries tend to be more productive and competitive, and they are wealthier and healthier than less innovative countries [4]. The outcomes of transformations in income and mortality levels are difficult to measure; however, some studies have shown that advances in science and technology have contributed to these changes in the twentieth century [5, 6]. The discovery of new vaccines, drugs, and the development of medical techniques have been some of the tools promulgated in the management of health crises, especially epidemics [7]. As a result, investment in research and development (R&D) is considered essential in the field of public health to create not only social value but also economic or monetary value. Moreover, in recent years, increasing investments have been made in various innovations, especially digital technologies, related to well-being and health [8].

In general, health innovation is often equated with therapeutic and medical advancements or even changes in the organization of health services [9]. This orientation is based on a techno-centric vision of innovation in which technologies are seen as an end in themselves, and there is no arrangement between technical and social values [10]. In this perspective, the management of health crises is carried out with a focus on finding punctual, reactive and technical solutions to remedy a specific problem without a systemic/holistic and proactive approach [11].

This paper contributes to reflection on the epistemological orientation of innovations in the field of public health. Taking the Covid-19 and Ebola crises as examples, the primary objective is to show how innovation in health is oriented towards a techno-economic paradigm. First, we discuss how this paradigmatic orientation poses challenges for public health goals, knowledge and actions. Then, we question the purpose and usefulness of innovations during health crises and their alignment with the main principles of public health. Based on different lessons drawn from the Covid-19 and Ebola crises, we propose the repositioning of public health innovation towards a social paradigm. By emphasizing the interaction between social and health dimensions in a perspective of social change, we conclude by highlighting the different roles public health can play so that innovations have more social value.

## Health innovation: the technological-based view

Research in the public health sector has been significantly oriented towards technology-centred innovations, with an emphasis on products and processes. In fact, innovations are seen, above all, as imperative to stimulating economic growth [12]. There are two ways of looking at innovation from an economic perspective. The first emphasizes the results generated by the introduction of a new product, process, or method [13]. This perspective places the economy above all other spheres of society while still being external to it [14]. Innovation is then understood as an asset of the economy or a form of capital. It is neither a social process nor a vector of human development [14]. The second way considers the social or organizational process produced by innovation [13]. In this approach, any innovation, whatever goal it pursues, is a social process and has imperative social effects [10, 15]. However, the means and objectives of innovation are not oriented towards the “social” but rather towards the development of a “market” [16–18]. Its purpose is economic, yet innovation can have social repercussions for populations [16] through the social use of the product or process [18, 19]. Also, the social dimension of innovation is seen in the public sector as a means of guaranteeing the efficiency and effectiveness of the new product or process [20]. This pattern of thought then places productivity and economic growth above the concerns of meeting the most urgent and important health needs of society [21].

Recently, innovations have been developed in the fields of biotechnology and pharmaceuticals to monitor, contain, or treat infectious diseases such as Ebola and the more recent Covid-19. Among these technological and medical innovations, some are proactive and others are much more reactive, including the development of rapid diagnostic tests, molecular diagnostic tests, handheld devices, field laboratories [22], face masks, face shields, and mobile robots [23]. Further, many new drugs and vaccines have been developed or are under development [24–26]. For instance, since the outbreak of Covid-19, 115 candidates for the development of a vaccine were listed in April 2020, and 78 of them are active and confirmed [27]. Also, digital health (e.g. artificial intelligence, telemedicine, Internet of Things, mHealth, apps, health analytics) has become prominent in the global market. As of 2019, it was valued at USD 106 billion by the Global Market Insights, and the growth of this industry will be reinforced by the ongoing Covid-19 pandemic [8]. However, the rapid development of vaccines and treatments, particularly for Covid-19, was not seen for the Ebola virus, which was discovered in 1976. Before the 2014–2016 West Africa Ebola epidemic, the scientific literature listed only four completed clinical Phase-I trials for the development of a vaccine against this disease [28]. It has only been since August 2014, when the WHO declared this epidemic a public health emergency of international concern, that the international community, in particular donors and renowned pharmaceutical companies, has started to be mobilized for the development of an Ebola vaccine [28, 29]. In 2014, 46 clinical trials were identified [29], which led to the first licensed vaccine called Everbo (rVSV-ZEBOV-GP) that has been marketed since 2019 for the prevention of Ebola [26].

Although some of these innovations are useful, many of them were not well-designed or evaluated based on core issues such as their effectiveness, efficiency, or relevance to ensuring the health of populations [30]. In fact, pharmaceutical and biotechnology companies often use a value-based pricing strategy in which valuable innovations are analyzed through a cost-benefit lens [31, 32]. Through this type of analysis, the economic value of innovation is privileged over that of social value because the costs and consequences of innovative options are appreciated in monetary terms [33]. However, this market-based consideration has some limitations in terms of estimating the value of health, quality of life, and the cost of illness and death [32]. This orientation poses many challenges for public health, in particular for the accessibility and sustainability of health innovations, especially for the most vulnerable populations. In addition, it highlights the importance of the consideration of equity and health determinants in all innovation processes as well as the given values and ultimate objectives of health innovations. Below, we discuss three fundamental issues of this orientation while drawing on examples from the Ebola and recent Covid-19 crises.

### Confinement in technological determinism: technical innovations as a quick fix

In the health sector, technological advances are usually considered beneficial to society. This technological determinism often prevails in times of sanitary crises. In fact, many organizations and governments have used the terms “technological innovation” and “science development” when referring to the fight against Covid-19 or, previously, Ebola. On the United Nations’ website, one can read headlines such as “The United Nations is banking on technological innovation to curb COVID-19” [34] or on the UNESCO website “Fighting COVID-19 through digital innovation and transformation” [35]. Around the world, hackathons are being organized to strengthen technical solutions for Covid-19; for example, the #WeVsVirus or #WirVsVirus campaigns launched by the German government in which 26,000 people participated or the #CodeTheCurve campaign started by UNESCO and other partners. Concerning the Ebola epidemic, the same tendencies were shown in the scientific literature. Indeed, the governments and technical partners’ responses were focused on emergency and medical strategies by prioritizing technical solutions to control the transmission of the virus (like medicalized burial, patient control, or the adoption of good washing and hygiene practices) [36–38]. These dominant discourses and practices of local and international authorities have repercussions on the progress of activities undertaken to counter the epidemic. This often contributes to strengthening existing social, political and cultural inequalities and can lead the failure of health public responses [38, 39].

In this vein, the enhancement of R&D in innovations is focused on medical devices that can be used to stop or reduce the transmission of the disease. For example, CAN 54.2 million in funding has been allocated for R&D related to Covid-19 through the Rapid Response Program from the Canadian Institutes of Health Research [40]. In June 2020, 100 research projects were launched in two general areas: medical and social/policy countermeasures. For a total investment of 55.3 million, 53 research applications were funded, with an investment of 37.6 million going toward the area of medical countermeasures versus 17.7 million for 47 projects related to social/policy countermeasures [41]. This substantial difference in investment demonstrates how technological or medical innovations are perceived as the solution for dealing with health crises. However, the origin, spread, transmission methods, and effects of infectious diseases are not solely linked to biological factors. Environmental elements, such as deforestation, growing urbanization, climate change [42], and socio-economic issues [43] are also relevant to the prevention of and response to future health crises. Thus, the concentration of funding on limited innovations can obscure other sectors and does not allow for a global and integrated approach. Some experts have even noted that the funding of only a few innovative perspectives in the case of Covid-19 can lead to collective failure since the chances of success of such an approach are minimal [44]. Responding to crises requires an intersectoral and integrated approach that goes beyond epidemiological and medical considerations. Moreover, the Ebola crisis enabled experts to point out that a lack of coordination was one of the weaknesses in the fight against the epidemic. Some of their recommendations are to set up intersectoral coordination mechanisms at global, national, and local levels as well as to integrate social sciences with medical and technological knowledge to overcome the challenges encountered in the implementation of public health actions [42].

In addition, the usefulness and relevance of the frantic development of technological innovations should be addressed in the public health field. Indeed, it is well-established that many innovations that are produced, introduced, and disseminated are not based on scientific evidence [33, 45]. There is a lack of knowledge of how widely evidence-based approaches are being utilized in the innovation process [33, 46] and how to promote the conception of more valuable innovations [47]. Innovations are usually made by specialists (an individual authority or collective epistemic authority) without compromise nor negotiations with the other actors concerned and without successive adaptations throughout the process [48]. Consequently, many new interventions fail to be adopted, spread, scaled up or sustained in the health care system and communities [49, 50]. The underlying reason for this is that many of the innovations adopted and disseminated in adoption systems have unproven effectiveness and show limited benefits for users [51]. Indeed, valuable innovations are not necessarily guided by values shared by different relevant actors (such as efficiency, cost-effectiveness, health system performance, equity, sustainability, etc.). As a result, many health innovations are marketed that neither benefit the entire population nor meet health needs [44, 47, 52].

Moreover, having R&D oriented based on market models contributes to an unequal distribution of the health benefits of innovation. In fact, there is a high possibility that a drug or a technology will be more rapidly developed for Covid-19 than for certain neglected tropical diseases or those deemed “unprofitable” [53]. In this regard, the World Health Organization (WHO) highlights that market-driven models of R&D do not promote the development of medical technologies for certain sporadic or unpredictable diseases, such as SARS, MERS, and Nipah virus infection, that have emerged over the past two decades [54]. Also, when such diseases occur in low-income countries, there is a greater challenge for medical technologies to emerge. R&D into certain tropical infectious diseases, such as malaria or leishmaniosis, that are the cause of high rates of mortality and morbidity in developing countries, are neglected since they do not provide sufficient financial income for pharmaceutical companies [32, 53]. In the case of Ebola, as highlighted above, the time between the discovery of the virus and the marketing authorization of the first vaccine took 43 years. Furthermore, the scarcity of personal access to effective and safe health services, products and technologies in the West African region could explain the failure of some of the responses to the Ebola epidemic, such as individual health security [55]. In fact, when oriented towards commercially viable targets, global investment in R&D greatly benefits developed countries that offer a viable market for technological innovations. This leads to R&D into diseases that affect the poor being neglected [53, 54]. For instance, in the case of Covid-19, in which the profit-driven component is favored, R&D projects are abundant and there is widespread competition in the same types of innovations, which could lead to unnecessary duplications. Considering this, there is a need for public health to adopt greater vigilance to better choose the innovations that are most likely to sustainably improve the health of populations, including the most vulnerable, and planetary health. Global public health must then ensure that the resources used serve to obtain the best possible result at the individual and collective levels [56].

### Innovate for whom? The misconception of innovation neutrality

Research and actions in the field of public health have largely turned to the dissemination of innovations. For some authors, diffusion is the key element in differentiating novelty from innovation [1]. Everett Rogers defined this as a process through which an innovation is communicated over time through the use of different communication or influence channels and adopted among members of a social system [1]. This perspective is based on the principle that innovation is a “finished concept”, which implies that, once theorized and conceived, it is enough to communicate and promote it for it to be adopted or to find a buyer. In other words, innovation is seen from a linear and mechanistic perspective that risks minimizing any sense of controversy, opposition, or even rejection that potential adopters might have towards it [48, 50]. However, all innovations do not spread spontaneously. Some innovations require the active involvement and concerted efforts of multiple stakeholders [57].

In this vision of the dissemination of innovations, little attention is paid to potential users and the setting-up and adoption contexts. In this sense, Green, Ottoson [58] note that the conception, development and implementation of innovations are done institutionally; in other words, the knowledge related to innovations is developed by scientists, then verified and disseminated by decision makers and practitioners. This type of functioning and reasoning comes from a purely biomedical perspective, in which evidence-based medicine justifies the implementation of an innovation whose effectiveness and efficiency are often reduced to the characteristics of the product or of the idea that is perceived as new. In this paradigm, innovations are approached from an economic perspective in which the idea, knowledge, technology, product, policy, process or practice that are perceived as new are designed and developed in R&D departments and then disseminated naturally within adoption and use systems [48]. As a result, an innovation is perceived as a novelty or invention, and it can greatly inspire health policies. In this regard, the political agendas of the western world favor the development of health innovations that propel new technologies and treatments with the political objective of creating wealth through R&D while optimizing the management of health care [21]. Thus, university entrepreneurship has become an important concept in the field of health innovations, which translates into a university-industry alliance oriented much more towards the market than towards the public interest [59]. These mechanisms are to a certain extent profitable for regional or even national economies [59]. However, this presents unavoidable challenges for public health, where the health and well-being of populations in a dynamic of equity and responsibility must be the main concerns [33].

The innovation is then decontextualized and implemented in a bureaucratic, vertical vision, which leaves little room for the involvement of multiple actors in the implementation and dissemination processes [60]. Moreover, some research has already pointed out that the responses that have been promoted to fight against Covid-19 and Ebola have been deployed in a top-down, paternalistic approach, allowing neither an intersectoral and interdisciplinary approach nor citizen involvement [30, 36, 38]. Some lessons learned from the Ebola crisis have shown that it is necessary to link effective top-down global responses to local actions [55]. In fact, the adaptation of measures is key to the success of innovation in times of crisis. In practice, however, the main focus is on innovative responses that are born and disseminated centrally or vertically to the detriment of those that emerge on the periphery of institutions. Also, many studies that have used the diffusion of innovation theory in the field of public health have focused primarily on behavioral theories, for example, by focusing on the adaptation of messages to the individual level and the use of change agents to influence or act on the potential users of an innovation [50, 61]. In this perspective, these studies have greatly minimized the adoption contexts and the conditions for the establishment, dissemination and sustainability of innovations [49, 58].

This may explain to some extent the observed failures of certain promising innovations in the health system or even resistance from users [52]. Indeed, the introduction of a new medical or technological discovery or even the establishment of a prevention system without the effective involvement of potential users may prove to be a failure. In the case of Ebola in the DR Congo, little effort has been made to understand the concerns of the population or their understanding of the messages transmitted by public health, which has contributed to resistance, ranging from the rejection of prevention activities to acts of violence [62]. Some studies have argued that technological development is beneficial for fighting health crises, but it is becoming more and more urgent to put in place mechanisms to build trust between communities and the health system [37, 55, 62]. Low trust in institutions and misinformation can significantly reduce adherence to preventive behaviours in the case of Ebola [62]. For example, in Guinea, isolating populations through Ebola treatment centers (ETCs) and the use of ambulances have contributed to mistrust and resistance from some communities. Indeed, these communities have concerns that ambulances are being used to infect populations intentionally and ETCs will steal certain parts of the human body [38]. However, the perspectives of these populations are not devoid of meaning; they are part of a universe of meaning and significance linked to local and global realities. Thus, it is important during sanitary crises that public health interventions recognize differences, even the most radical, and not obscure the history of structural violence in some communities [38]. Also, the consideration of local cultural practices such as burials, religious groups, and community-based organisations is an important part of renewed trust-building [55].

On the other hand, the control of epidemics is often accompanied by coercive methods that reinforce the fear of communities and their distrust of public health measures. For example, restrictive measures have been taken in the case of Covid-19 and Ebola such as quarantines enforced by the police (even the army), rigid lockdowns, isolation, medical surveillance, and sanitary cordon. Thus, the coercive pandemic response could work initially, but with decreasing trust in institutions, the response could be undermined in the medium term [63]. To effectively fight against epidemics, public health can no longer rely solely on the development of innovations “out of context”, and it is necessary to engage and empower citizens and communities in the development and deployment of health innovations. The access of citizens to scientific knowledge must be improved, especially in times of crisis [64], as well as the integration of their knowledge in the innovation process [30]. This is becoming more and more important in the current global context, in which populations are very distrustful of health interventions and fearful of certain technological or medical advances such as vaccines. Concerns have even been raised about the rise and rapid spread of rumors and misinformation related to Covid-19, which the UN has termed “Infodemia” [65], especially in certain vulnerable populations or those experiencing social inequality [66].

Consequently, to build trust during a health crisis, some relevant lessons from Ebola could be useful for the future, such as the implementation of community engagement strategies that give communities a voice and allow them to be heard [42]. In addition, the use of communication channels and methods that people already know and use (e.g. social media, community radio) and maintaining transparency and consistency in the responses to local needs could help build trust [42, 62]. Whether it is a bottom-up or top-down process, it remains fundamental to consider that innovation can take different forms of partnership (between companies, associations, administrative institutions, professionals, volunteers, or others). Ensuring a balance of power and knowledge between different actors is necessary to create social value. As innovations are mainly initiated and supported by public authorities (by financing, regulating or coordinating them), it remains imperative to combine the strengths of top-down and bottom-up approaches [67]. Also, governance is a useful point to consider, because the success of an innovation may depend in part on favorable support from political, administrative, and governance institutions [68]. For these reasons, it is increasingly urgent to facilitate transformation in the global health governance system, which is fragmented by multiple players that implement parallel top-down programs without positive repercussions on health systems [11, 55]. For proactive public health during an epidemic, it is necessary to prioritize R&D that is tailored to unmet health needs and mechanisms that will favor the development of responses that are relevant, safe and at a lower cost for users [55].

### Equity issues facing market laws

As previously pointed out, innovation from a techno-economic perspective is largely influenced by economic standards and values, which risks overshadowing major public health issues, in particular the reduction of social and systemic health inequalities. This issue remains crucial, especially in times of crisis. In fact, there is an unequal distribution of the risks posed by disease, as they are concentrated in the most socially vulnerable, which has been widely noted in the Covid-19 pandemic [69, 70]. Recent data have shown that socially vulnerable people are unequally affected by Covid-19. They are the most exposed and at the highest risk of developing the disease, and they suffer more severe consequences of the disease. They also have poor access to high-quality public health and medical care. For example, in the United States, black Americans living in environments marked by social injustice, structural racism, and poverty die the most from Covid-19 [70]. In addition, public health responses, such as social distancing or the closure of schools or non-essential services, can generate negative impacts on psychosocial health, social isolation, family relationships, behavior related to health, and education among the most socially and materially disadvantaged [71]. In this regard, the invisibility of women and gender during the Ebola and Covid-19 crises is discussed in the literature [39, 72, 73]. Based on the experience of the Ebola epidemic, Sophie Harman [73] raised the conspicuous invisibilization in global health policy and practices of the different impacts of the disease on women and men and the particular role of women as family caregivers and frontline health professionals in West Africa. This “gender-blind” perspective could lead to irrelevant and potentially stigmatizing public health communication [39] and could maintain or reinforce gender inequity.

In this perspective, some authors have criticized the fact that artificial intelligence has been limited only in its technical possibilities in public health [56, 74]. As a result of this simplistic vision, its utilization has not taken into account questions related to health equity and how social determinants could be addressed [56, 74, 75]. Public health interventions during sanitary crises are increasingly based on the use of digital technologies. For example, communication and awareness-raising activities are carried out through the internet and social media, yet there are inequalities in the access to and use of digital technologies [76]. As a result, responses that attempt to address the current crisis can exacerbate pre-existing inequalities and contribute to making certain groups more vulnerable [76]. In addition, crises are often times when the price of essential commodities or services can rise; therefore, access to health services and products during a pandemic is a priority internationally. The case of face masks during the Covid-19 crisis is particularly striking. Despite being a compulsory measure in several developing countries, it has been reported that many people do not have access to face masks because of their price. In some places in South Asia, a face mask can be priced as high as USD $7 [77]. For this reason, several organizations, such as Doctors Without Borders, are calling for technological and biomedical innovations to be developed with the help of government funding to serve the interests of public health [78].

Also, certain non-pharmaceutical measures taken during the Covid-19 crisis, such as confinement, social distancing, hand washing with appropriate facilities, consulting a doctor promptly in case of symptoms, and obtaining information are considered difficult to carry out for many people living in poverty [79, 80]. As a result, some authors have suggested that the preventive measures proposed by the WHO and governments are modeled on the lifestyles of wealthy countries, and their relevance is questionable in certain contexts of developing countries [80]. This reinforces the knowledge that effective innovations too often benefit certain populations, while others do not have access to them [81]. In addition, innovations should help reduce existing inequalities and not contribute to exacerbating them. This emphasizes the importance of public health in committing to adopt responsible innovations based on fundamental principles such as relevance, efficiency, equity, and social justice for rapid and lasting responses [30]. In a vision of sharing benefits and equitable access, it is important to implement innovations that can reduce health inequalities by putting the social and systemic determinants of health approach at the heart of the process. In addition, sustainable innovations that do not damage or affect personal, public, and planetary health are necessary. For that, in a dynamic of proactivity and sustainability, it is relevant to put forward an integrated and eco-social approach to health in which ecological determinants are complementary to the social determinants of health [82]. In this sense, the innovations promoted in public health must take into account their relationships with and consequences for the various elements related to ecologies and ecosystems at local and global scales.

In the following sections, we will offer a new perspective on innovations in public health. We align ourselves with the idea that innovation and health are both social constructs [9]. R&D should emphasize the interactional dimensions between different values of innovation, such as economic and social values. It remains necessary to integrate technology into culture [17] while considering innovation as a multidimensional process that combines several organizational, technological, economic, and social arrangements. In line with this, considering the social and systemic determinants of health, intersectoral collaborations are necessary to tackle new challenges in the health sector, especially during sanitary crises [30]. As part of this momentum, the human should be at the center of concerns. In other words, understanding health in a holistic way should be the focal point of systems, policies, and research in the field of public health. In relation to this, Table 1 presents a global vision of the existing differences between the techno-economic and social paradigms of health innovation.

**Table 1:**
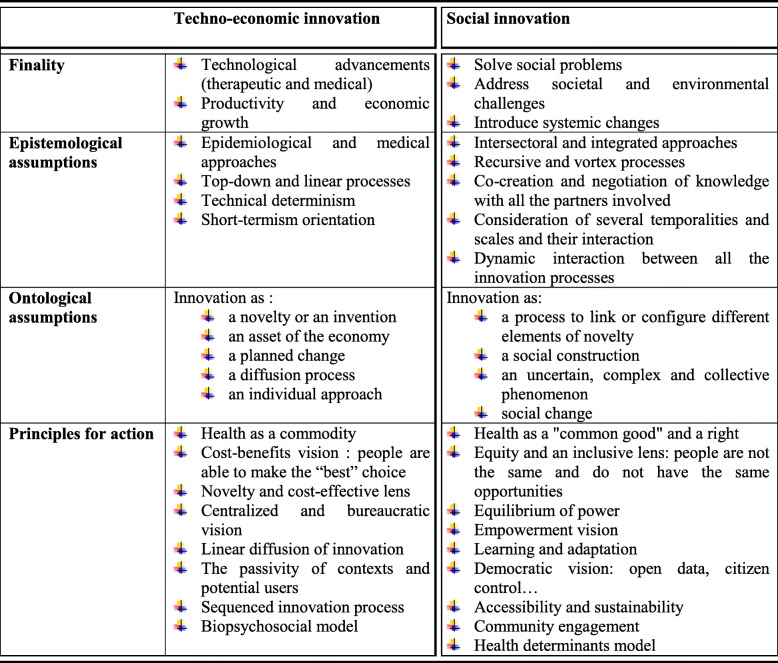
Differences between the techno-economic and social paradigms of health innovation

## Conclusion: social innovation as a paradigm shift in public health

In the argument presented above, we have demonstrated how public health responses to health crises, Ebola and Covid-19 in particular, have been mainly oriented toward a techno-economic paradigm. This orientation has two main repercussions on: (1) how individuals and communities receive and respond to the public health actions undertaken and (2) the health and well-being of individuals and communities. These elements have enabled us to understand that social and environmental determinants and health equity, which are important issues in public health, have thus been largely ignored in public health actions. Indeed, the various examples drawn from the Covid-19 and Ebola health crises have shown that public health actions have mostly focused on emergencies instead of taking a preventive and health promotion lens, which has prevented the effective consideration of social inequalities in health. In this sense, we point out that the lack of consideration of social and environmental determinants contributes to exacerbating structural violence and also to promoting social resistance in the fight against the current pandemic.

Overall, the various issues raised here demonstrate that innovation in the field of public health should not be seen simply from a techno-centric perspective. Indeed, it is also not a single-person concern. Innovation should not be guided only by market logic; it must be accepted by and accessible to a large number of people and must also meet a real need. Innovation does not necessarily derive from the conception of a “new” idea or product; it is a process that makes it possible to link different elements of this novelty. For example, any invention, idea or technology is not necessarily an innovation that is expected. Therefore, the social process deployed in its use within systems remains important. The value of public health innovation is rooted in its accessibility to everyone, thus it responds to the right to health, but also improves the health of individuals and populations. In line with this, a public health innovation can generate different forms of value, not only economic.

In this perspective, from the Ebola and Covid-19 epidemics, we can draw some useful lessons for public health in the management of ongoing and future health crises. Firstly, it is important to shift techno-economic innovation in the social innovation paradigm in public health. Indeed, in scientific literature, social innovation can be recognized by its results and objectives that focus on social goals. The latter must be oriented towards improving the well-being or the quality of life of individuals, communities [83], and the entire planet.

It is important to understand what “social” means in the process of innovation, considering that technological innovations can generate social benefits that are not their primary objective [18]. What social value can social innovation produce that may be different from that generated by technological innovation?

Hubert [84] proposed three social goals for social innovation. First is the resolution of pressing social problems, such as the exclusion and integration of vulnerable people, health problems, and the organization of care, by focusing on social relations between individuals. Second is addressing societal and environmental challenges by transforming the social relationships, norms, and social and cultural representations shared in a society [83, 85]. Finally, systemic change should be introduced by contributing to the reform of systems or sectors of society in which participation, empowerment, and learning are the sources and ends of well-being [86]. These three approaches must be considered in a complementary manner to provide effective and lasting solutions to the problems facing our current societies [86, 87]. These three goals are useful to consider during a sanitary crisis that requires swift action to limit the spread of the disease and engage in innovative, proactive, and systematic approaches. In addition to biomedical measures, public health interventions must take into account various elements such as environmental, economic, social, religious, and cultural issues to tackle contemporary societal challenges. For that, the participation of all relevant actors and organizations affected by the innovations is important to be able to set up lasting interventions that are useful and relevant for the immediate crisis and for the post-crisis. The process of innovation therefore remains a fundamental element that facilitates a better understanding of social innovation, both in terms of its means and operationalization.

Secondly, to create social value, public health actions should not be thought of and implemented from the perspective of linearity but of recursion. In this sense, in Table 2, we propose some roles that public health could play so that innovations can have more social value, especially during sanitary crises.

**Table 2:** Public health roles during sanitary crises for generating social value

1. “Do not harm”: remain vigilant and do not exacerbate social inequalities by considering equity in all actions taken. Assess the potential risks of innovation on population and planetary health before implementation. 2. “Do not let stigmatization arise”: adopt non-stigmatizing and inclusive communication and sensitive communication mechanisms for existing inequalities (literacy, access to digital tools, allophone populations, etc.). Set up targeted communication approaches and sustainable popular education mechanisms and involve local and community institutions to prevent the spread of fake news. 3. Improve R&D funding: enhance the funding schemes for innovations that emerge on the periphery of institutions and set up funding mechanisms to combat emerging diseases in poor areas. Establish some main principles at the level of all the actors responsible for R&D projects financed by public authorities such as co-creation with the partners involved, citizen participation, a vision of the social determinants of health, and the imperative that innovation meets the real health needs of communities. 4. Improve the sustained funding and visibility of prevention and health promotion systems in different domains such as health mental, suicidal behaviour prevention, and familial violence. 5. Build bridges between institutions and citizens: stimulate citizen participation through mechanisms that can bring public authorities and citizens closer together and strengthen the bonds of trust in society. Promote citizen participation and social mobilization by using existing community networks and resources while promoting civic education and civic literacy. 6. Contextualize and adapt emergency public health measures to different localities: this should be based not only on epidemiological measures, but also on social, cultural and political realities. 7. Set up mechanisms for sustainable and secure coordination and collaboration for different actors and areas of expertise and at different scales (global, national, regional, and local). 8. Ensure the accessibility (financial, geographic, and cultural) of the health innovations developed. 9. Plan and frame actions according to a temporal logic that takes into account not only emergency measures but also long-term measures.	

Finally, we propose that public health should embrace complexity theories and approaches that allow innovations to generate social values. In recent years, several frameworks have emerged for facing complex problems and current societal challenges, such as Wicked policy problems [88] and One Health [89], among others. Therefore, we call upon public health actors to adopt complexity as a way of thinking and acting that allows the consideration of relationships, interdependencies, disorder, order, emergence, unforeseen circumstances, and recursion as phenomena inherent to the innovation processes.

## Data Availability

Not applicable.

## Abbreviations

R&D: Research and Development
WHO: World Health Organization
